# I Luso-Brazilian Positioning on Central Arterial Pressure

**DOI:** 10.5935/abc.20170011

**Published:** 2017-02

**Authors:** Andréa A. Brandão, Celso Amodeo, Cristina Alcântara, Eduardo Barbosa, Fernando Nobre, Fernando Pinto, José Fernando Vilela-Martin, José Mesquita Bastos, Juan Carlos Yugar-Toledo, Marco Antônio Mota-Gomes, Mario Fritsch Toros Neves, Marcus Vinícius Bolívar Malachias, Manuel de Carvalho Rodrigues, Oswaldo Passarelli Junior, Paulo César B. Veiga Jardim, Pedro Guimarães Cunha, Rui Póvoa, Teresa Fonseca, Vitor Paixão Dias, Weimar Sebba Barroso, Wille Oigman

**Affiliations:** 1 Departamento de Hipertensão Arterial da Sociedade Brasileira de Cardiologia, Rio de Janeiro, RJ - Brazil; 2 Sociedade Portuguesa de Hipertensão- Porto - Portugal

**Keywords:** Arterial Pressure, Cardiovascular Diseases/physiopathology, Coronary Diseases/physiopathology, Risk Factors, Endothelium, Vascular, Atherosclerosis

## Natural and accelerated vascular aging. Involved mechanisms and factors

### The vascular aging process

In 2006, Dzau et al. presented the cardiovascular disease (CVD) continuum, represented by successive events/stages of disease progression from the incidence of known risk factors until death.^1^ This whole concept had the genesis and progression of atherosclerosis as its nuclear mechanism of progression to underlying CVD. In 2010, Dzau et al. gave new emphasis to the importance of age-related structural changes in the middle layer of the arterial wall (arteriosclerosis) as a contributing mechanism for the risk of development of CVD.^2^

There is a natural process of wear and progressive modification of the arterial wall structure that arises from the mechanical stress of distension induced at each cardiac cycle in connection with the pulse wave amplitude and incident and reflex pressure.^3^ In the absence of any other factor, this mechanism alone will produce wear on the arterial wall, promoting thickness reduction, fragmentation, and disorganization of the elastin layers. In parallel, this damaged elastic component is replaced by collagen and protein matrix, which is less capable of accommodating the incident pulse wave pressure. In addition, there is loss of integrative and functional connection between elastin layers and smooth muscle vascular cells,^4^ resulting in reduced distensibility and increased stiffness of the large artery wall, which can be measured by an increase in the transmission of the pulse wave velocity (PWV) and the return of the reflex wave. Thus, there is an influence on the central systolic blood pressure (cSBP), central pulse pressure, "augmentation index", and other ventricular-vascular integration indices.^5^

The factors accelerating arterial aging are multiple: fetal programming, genetic factors, hypertension, dyslipidemias, diabetes mellitus, chronic renal disease, chronic diseases with an inflammatory component, and smoking, among others.

### Accelerated vascular aging

The identification of individuals with accelerated vascular aging may allow an earlier specific intervention, with control of the various risk factors. For each carotid-femoral PWV (cfPWV) increase of 1 m/s, the risk of cardiovascular death, cardiovascular event, or mortality from other causes increases between 14 and 15%.^6^ The publication of cfPWV^7^ reference values for different age groups has allowed an easier identification of individuals with early signs of arterial stiffness. However, ethnic and/or environmental exposure aspects that may also contribute to the arterial aging process should be taken into account in the definition of "normal".^8^

### Arterial aging: relationship between microcirculation and macrocirculation, and between arteriosclerosis and atherosclerosis

We can identify four key milestones in the vascular aging process: 1) a progressive reduction in the distensibility of large muscular arteries; 2) a progressive increase in the reflected pressure wave, with a consequent increase in the various components of central arterial pressure; 3) a loss of the arterial stiffness gradient between the central and peripheral arteries; and 4) a progressive elimination of the impedance differential between the arterial macrocirculation and microcirculation.^9-11^ This set of structural and functional changes in the arterial tree following the deterioration of the structure and function of the middle layer of the arterial wall (arteriosclerosis) is associated with the appearance and concomitant development of atherosclerosis lesions in the vessel wall, having endothelial dysfunction as a unifying mechanism.^12^

## Measures of Central and Peripheral Pressures: Differences and Advantages

**Figure 1:**
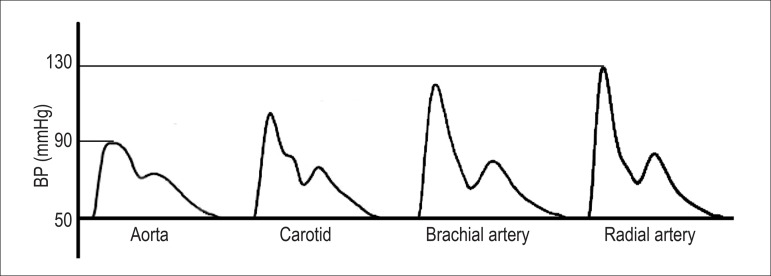
Amplification of systolic pressure from central to peripheral arteries.

Brachial blood pressure (BP) measured with a sphygmomanometer cannot be considered equivalent to aortic pressure since the latter has invariably lower values. The BP varies continuously during the cardiac cycle, although in practice only the maximum value during systole and the minimum value during diastole are measured. Furthermore, the shape of the pulse wave varies along the arterial tree. With the advancement of the pulse wave from the more elastic central arteries to the more rigid peripheral arteries, the systolic peak becomes narrower and more elevated (Figure 1). Considering that the diastolic BP (DBP) and the mean BP are relatively constant, the brachial systolic BP (SBP) can be 30 mmHg higher than the central systolic aortic pressure in young individuals. This phenomenon, known as amplification of systolic pressure (or pulse pressure), occurs due to several reasons, among them the smaller caliber and greater stiffness of the peripheral arteries. In addition, pulse wave reflections occur at several sites in the arterial network, such as areas with greater collagen/elastin gradient, with greater vasomotor tone and, especially, at bifurcation points. Multiple reflected pulse waves integrate into a single reflected wave that is added to the incident pulse wave, caused by the ventricular ejection. When the reflected wave reaches the incident wave earlier, there is an increase in the central systolic pressure and, consequently, a reduction in the amplification of the pulse pressure. In fact, this increase in pressure depends on several variables, especially age, gender, height, and heart rate.^13-15^ Female gender, advanced age, short stature, and bradycardia are associated with a lower pulse pressure amplification. Even with the control of these variables, only about 70% of the variability in the pulse pressure amplification can be explained in multiple regression models.^13,16^ This indicates that central pressure cannot be accurately estimated from the brachial pressure using statistical models, but it actually needs to be determined directly through appropriate methods.

### Advantages

Measurement of central pressure could result in greater accuracy in the diagnosis of hypertension, greater safety in the therapeutic decision, and better definition of the prognosis.^17,18^ Some authors have identified that central pressure, compared with brachial pressure, correlates better with intermediate cardiovascular risk markers such as carotid intima-media thickness and left ventricular hypertrophy.^19,20^ Several studies have reported an independent relationship between central pressure and future cardiovascular events, including in elderly patients with coronary disease and chronic kidney disease.^14,21-23^ However, other studies have not found a superior predictive value for central pressure over brachial pressure.^24^ This controversy exists because the methodology is still heterogeneous and the peripheral pressure, necessary for the final result, explains more than 90% of the variation in central pressure. Furthermore, derivation of central pressure requires an additional measurement, usually radial tonometry, which is also subject to errors that may contribute to the remaining 10% of the variation.^18^ Therefore, before recommending central pressure measurement for wide clinical use, standardization of the method and the calibration system, and technical limitations of the various devices available must be resolved.

## Definition, evaluation and normal values of the main central parameters (central aortic pressure and carotid-femoral pulse wave velocity)

The SBP values vary considerably according to the place where they are obtained. The SBP is greater in the brachial artery when compared with the aorta. This difference in pressure values between the aorta and the brachial artery is a consequence of the phenomenon of peripheral amplification, which results from the difference in impedance between the large-, medium- and small-caliber arteries, especially in the bifurcations, and also the presence of several factors of interference, such as age, comorbidities (dyslipidemia, smoking, diabetes mellitus, etc.) and environmental factors (sodium).^25^ Recent evidence indicates that central aortic pressure, the augmentation index, and cfPWV are robust markers for future cardiovascular events.^21,26^

An important aspect in relation to central systolic pressure concerns pressure values obtained with commercial equipment by noninvasive methods. Although these values correlate well with invasive studies, they do not fully represent the central systolic pressure values, but they correctly reflect the amplification phenomenon. This static measurement is considered insufficient for a definitive validation of these methods in the stratification of cardiovascular risk.^27^

Measurement of cfPWV is an appropriate method of assessing arterial aging with an excellent correlation with the risk of cardiovascular death, cardiovascular events, and mortality from other causes.^6^ The stiffening of the distal aorta and large arteries, such as the carotid and iliac arteries, occurs due to the early return of the reflection wave, secondary to structural and functional alterations of the distal vascular wall.

Therefore, the great arteries differ from medium and small arteries in relation to histology, physiology, and elastic properties, which is why it is extremely important to define the anatomical target for the action of a drug and the therapeutic target to be achieved. Evidence regarding drug treatment points to a greater ease of reversal of alterations in small-caliber arteries (muscle) than in large arteries (elastic). Thus, results obtained in one arterial segment cannot be extrapolated to other segments in the same arterial tree. Tables 1 and 2 show the central aortic systolic pressure, augmentation index, and cfPWV values in the normal population.^28,29^

**Table 1:** Central systolic aortic pressure values and the augmentation index in normal individuals^28^

	Central aortic pressure (mmHg)				Augmentation index (%)			
	Female		Male		Female		Male	
Age (years)	Mean	Percentile (10-90)	Mean	Percentile (10-90)	Mean	Percentile (10-90)	Mean	Percentile (10-90)
<20	97	86 -109	105	96 - 113	14	9 - 20	19	11 - 24
20 - 29	95	80 - 110	103	92 - 115	12	5 - 19	15	6 - 24
30 - 39	98	84 - 119	103	88 - 120	8	0 - 17	13	4 - 23
40 - 49	102	87 - 123	106	90 - 123	6	0 - 15	11	2 - 21
50 - 59	110	93 - 127	110	96 - 126	5	0 - 13	9	2 - 18
60 - 69	114	97 - 129	114	97 - 128	6	1 - 12	8	2 - 17
> 70	118	100 - 131	116	99 - 130	6	1 - 13	8	1 - 17

% = percentage increase.

**Table 2:** Carotid-femoral pulse wave velocity values (m/s) in normal individuals^29^

Age	Mean ± 2SD	Median (percentile 10 - 90)
<30 a	6.6 (4.9 - 8.2)	6.4 (5.7 - 7.5)
30 - 39 a	6.8 (4.2 - 9.4)	6.7 (5.3 - 8.2)
40 - 49 a	7.5 (5.1 - 10.0)	7.4 (6.2 - 9.0)
50 - 59 a	8.4 (5.1 - 11.7)	8.1 (6.7 - 10.4)
60 - 69 a	9.7 (5.7 -13.6)	9.3 (7.6 - 12.1)
> 70 a	11.7 (6.0 - 17.5)	11.1 (8.6 - 15.5)

SD: standard deviation.

## Evaluation methodology - available devices and their validations

The cfPWV, which directly reflects arterial stiffness, has a predictive value in cardiovascular morbidity and mortality and is currently considered the gold standard method to assess arterial stiffness.^5^

The devices used to measure cfPWV have evolved over the last two decades, and their new versions have received systematic validation. Numerous studies have been published comparing invasive and noninvasive methods in different populations and among several existing noninvasive cfPWV measurement devices such as oscillometric, piezoelectric, and tonometric. Most of them have a good correlation with the methods most used in epidemiological studies, such as Complior® or SphymoCor®, among others. Currently, these methods involve little operator training and the ease of use and time consumed in the exam have been optimized so that they are becoming more available for use in clinical practice with good intraobserver and interobserver correlations.^30^

Some differences have been found in studies comparing devices, with higher values ​​of systematically hemodynamic parameters obtained with one device in particular. The mathematical models used in different devices can lead to different results. However, in most cases, this has no translation in clinical practice, since it does not imply a change in the risk class of the individual. Nevertheless, it is prudent that the same type of equipment is used in multicenter research studies.^31^

In addition to the validation of different equipment, different procedures for measuring cfPWV have also been proposed. These different procedures, such as measuring the carotid-femoral distance, can influence the results obtained if they are not also standardized. In this case, there are arguments that 80% of the direct carotid-femoral distance is the most accurate estimate for this same distance.^5^

## Central parameters: differences according to age, sex, and ethnicity

The best way to define normal values for central aortic pressure would be a correlation between the central aortic pressure levels obtained and the cardiovascular risk, as known for the BP obtained by the conventional or brachial method. However, these data are not yet available as results of prospective studies designed for this specific purpose, although some publications have sought to obtain these correlations between cardiovascular outcomes and central aortic pressure.^21^

One strategy would be to obtain correlations between central aortic pressure values that correspond to conventional pressure values obtained in the casual brachial artery or in the clinic. Following this strategy, population studies suggest that an optimal systolic central aortic pressure would be represented by values < 110 mmHg, which would be equivalent to 120 mmHg when obtained by the conventional BP measurement. Likewise, a central aortic pressure < 120 mmHg would correspond to a brachial SBP of 140 mmHg, defining as stage 1 systemic arterial hypertension a systolic central aortic pressure ≤ 120 mmHg.^32^

## Applicability and cost-benefit relationship of the measurement of central parameters

Although it is not part of the stratification routine in hypertensive patients, the central aortic pressure has attracted increasing interest due to its predictive value for the occurrence of cardiovascular events, as well as for the differential evaluation of the different anti-hypertensive drugs, when compared with the traditional determination of the brachial pressure.^33^ The augmentation index and the pulse pressure measured by carotid tonometry have been considered independent predictors of cardiovascular mortality in end-stage renal disease. However, the predictive value of the central aortic pressure, when compared with that of the brachial BP showed no significant differences.^21^ Nevertheless, the recommendation for its routine use requires further studies. As an exception and as an added value, isolated systolic hypertension is observed in youths, since the brachial artery SBP in these individuals may be increased due to an exaggerated amplification of the central pressure wave, which would be normal.^34^

There are no data verifying the cost-benefit relationship of central aortic pressure determination, extrapolating it from small studies with the use of angiotensin II receptor blocker (e.g., losartan), which reduces central aortic pressure and may bring some additional benefit when using it in addition to the reduction of brachial BP.^35^

## Isolated systolic hypertension in young adults: true hypertension and spurious hypertension

The pathophysiological mechanism of isolated systolic hypertension in elderly and young individuals is not the same. In addition, information on the prognosis of both is scarce and the guidelines currently available offer different recommendations on how to address these situations depending on the age group.^34^

Isolated systolic hypertension in young adults (ISHY) was described in 1999 as a "spurious" elevation of the SBP or pseudo-elevation of the SBP (> 140 mmHg) with normal values ​​of diastolic pressure (< 90 mmHg) resulting from a phenomenon of amplification of the peripheral arterial pulse waveform.^36^ ISHY is more common in male athletes, in individuals who are taller, and in those with higher body mass index.^37^ The prevalence of ISHY shows a significant variation (between 2% and 16%) in exclusively male cohorts and has obesity and tobacco as two of the main determinants.^38^ The noninvasive evaluation of the central pressure and pulse wave amplification in the upper limbs has a precise indication in these cases, since it allows the identification of young adults with "spurious" isolated systolic hypertension, sparing them from being labeled as "hypertensive patients".^39^ The identification of patients with ISHY should be complemented by outpatient monitoring to exclude white coat hypertension.^40^

ISHY has increased in prevalence and, given the lack of information about it, there are controversies about how to intervene in this situation. If on the one hand the values of central aortic pressure in individuals with ISHY are lower than those found in true hypertensive patients, they are higher than those obtained in normotensive patients.^39^ The study by Yano et al.,^41^ of 2015, showed a higher cardiovascular risk in this group when compared with individuals with optimal BP, but the study did not include an assessment of the central pressure for a possible differentiation between the groups.^41^ With the information available, the management is to carefully monitor with nonpharmacological measures, with a more aggressive management reserved for situations of greater associated cardiovascular risk, at least until new data are available.^42^

## Prognostic value of the ambulatory arterial stiffness index

The ambulatory arterial stiffness index (AASI) is used for the evaluation of arterial stiffness and is calculated based on the slope of the diastolic pressure *versus* the values from the systolic pressure in outpatient monitoring, evaluating the dynamic relationship between the DBP and the SBP in 24 hours.^43^

Thus, for any increase in the distension of the artery wall, the SBP and DBP values tend to increase in parallel, whereas in a rigid artery, there is an increase in the value of SBP accompanied by a lower elevation or even a decrease in DBP. Li et al.^44^ confirmed in a healthy Chinese population that there was a significant correlation coefficient between AASI and cfPWV, which is the gold standard method.^44^

The AASI depends on the degree of functional and structural integrity of the arteries, and may also depend on the ejected systolic volume and the reflection waves.^45^

Because the AASI is dependent on the mechanical properties of small arteries and reflection waves, this index correlates well with pulse pressure and augmentation index, and has a good correlation with some markers of lesion in target organs (ventricular hypertrophy, carotid lesion, and microalbuminuria).^46^

Some studies have shown a relationship between AASI and global and cardiovascular mortality, as well as a relationship with stroke in normotensive individuals.^47^ Nevertheless, this prognostic value is still debatable and is related to the degree of decrease during sleep and other factors, such as heart rate and peripheral vascular resistance.^48^ Moreover, its reproducibility is poor (around 50-68%).^49^

## Central parameters as predictors of arterial hypertension

There is evidence that increased arterial stiffness is a precursor to the occurrence of hypertension and not a consequence of increased BP. The increase in cfPWV preceded the appearance of hypertension over 7 years in an analysis of the Framingham Heart Study.^50^ The Baltimore Longitudinal Study of Aging also demonstrated an association between increased cfPWV and a higher incidence of hypertension.^51^ Other central parameters emerged as predictors of hypertension, such as increased brachial-ankle pulse wave velocity, increased proximal aortic stiffness assessed by echocardiography, and increased carotid artery stiffness, as demonstrated in the Atherosclerosis Risk in Communities (ARIC) study.^52-54^

The increase in aortic stiffness correlated with a lower sensitivity of the baroreflex, a precursor mechanism for the development of hypertension, as well as an increase in the BP variability.^55,56^

## Central parameters and cardiovascular risk

### Role of the carotid-femoral pulse wave velocity as a predictor of cardiovascular outcomes

The cfPWV is the most studied central parameter; consequently, there is a greater amount of evidence related to this parameter. Thus, it has been demonstrated that the cfPWV has an independent predictive value for different cardiovascular outcomes in different subgroups, as in patients with hypertension, type 2 diabetes, elderly and in those with end-stage renal disease.^57^ Even in apparently healthy individuals, cfPWV is an independent predictor of coronary disease and stroke.^58,59^ When the predictive values for cfPWV and peripheral pressure have been compared, the cfPWV showed an infallible superiority.^60^ A systematic review including 16 studies with 17,635 participants revealed that for each increase of one standard deviation in cfPWV, the risk ratio was 1.35 (95% confidence interval [95%CI] 1.22 - 1.50, p < 0.001) for coronary disease, 1.54 (95%CI, 1.34 - 1.78, p < 0.001) for stroke, and 1.45 (95%CI, 1.30 - 1.61, p < 0.001) for cardiovascular disease. These risk ratios were even higher in younger participants and remained significant even after adjustment for the presence of conventional cardiovascular risk factors.^59^

Small studies have shown that the persistent elevation of pulse wave velocity during the treatment of hypertension or cardiovascular disease is associated with a high risk for a cardiovascular event.^60^

### Role of the carotid-femoral pulse wave velocity in the stratification of cardiovascular risk

Studies have shown that the addition of cfPWV to traditional risk factors involved in scores such as Framingham and SCORE, and even atherosclerosis measures, significantly increases the predictive value for cardiovascular outcomes.^61-64^ They also indicated that cfPWV aggregates information for the stratification of cardiovascular risk, with the potential for clinical applicability. The use of cfPWV allowed to reclassify the cardiovascular risk range of the individuals and was able to improve the evaluation of the prognosis of cardiovascular risk in 10 years in individuals with intermediate risk by 13%, according to a recent systematic review.^59,65^ Thus, the presence of an elevated cfPWV measurement added to classic risk factors indicates an excess of cardiovascular risk and suggests the need for a more rigorous multifactorial approach.

### Role of central aortic pressure as a predictor of cardiovascular outcomes

One of the first publications to draw the attention of the scientific community to the role of central aortic pressure in cardiovascular outcomes, regardless of the peripheral BP values, was the Conduit Artery Function Evaluation (CAFE) study in 2006. In this analysis, the hypertensive patients who presented a greater reduction of the systolic component of the central aortic pressure to the same level of reduction of the BP values ​​obtained by the conventional evaluation had a lower incidence of cardiovascular outcomes.^66^ In that same year, the European Society of Cardiology published a position drawing attention to the fact that brachial measurements overestimate the central BP values ​​and that the systolic component of central aortic pressure, as well as central pulse pressure, are better predictors of cardiovascular outcomes, especially in patients with hypertension and chronic kidney disease.^37^ Other publications have also drawn attention to this superiority when comparing central measurements with brachial ones obtained from ambulatory BP monitoring (ABPM).^67^ On the other hand, a meta-analysis of 11 longitudinal studies showed that both central aortic systolic pressure and central pulse pressure were independent markers of outcome and cardiovascular mortality, but were not superior to the values ​​obtained by conventional measurement (peripheral pressure assessment, p = 0.057).^21^

## Relationship of central parameters with target-organ lesions and associated clinical conditions

Numerous studies have demonstrated that central BP measurement is promising in terms of better correlation with cardiovascular events.^68^ Differences in central and peripheral arterial pulsatility are difficult to be attributed to cardiovascular events.^69^ No studies have so far demonstrated robust evidence that central BP adds a new model of cardiovascular risk stratification in relation to the conventional SBP and DBP measurement. A recent analysis of data from the Framingham Offspring Cohort^70^ demonstrated a strong correlation between central aortic pressure and the incidence of cardiovascular events. A follow-up of up to 6.8 years in a population of 2,492 individuals (mean age 66 ± 9 years) has shown that 6% had a cardiovascular event. In a multivariate analysis, the measurement of central aortic pressure in this population correlated significantly with cardiovascular events. The CAFE study^66^ recruited 2,199 patients from the five centers of the ASCOT study and performed tonometry by radial artery applanation for analysis of central BP and pulse wave. Although the two arms of the study presented similar brachial pressure reduction (difference of 0.7 mmHg, 95%CI 0.4 - 1.7, p = 0.2), there was a reduction in central aortic pressure with statistical significance in the group that used amlodipine (central aortic systolic pressure 4.3 mmHg, 95%CI 3.3 - 5.4, p < 0.0001; and central aortic pulse pressure 3.0 mmHg, 95%CI 2.1 - 3.9, p < 0.0001). A *post hoc* analysis of this study demonstrated that central BP was significantly associated with combined cardiovascular outcomes and the development of renal failure (p < 0.05).

## Implication of the central parameters in the strategy for the treatment of hypertension

Despite the adequate reduction of (peripheral) BP with anti-hypertensive treatment, the results on clinical outcomes have shown a significant difference attributed to the pleiotropic effects of anti-hypertensive drugs on the elastic properties of large arteries (aorta), on the central aortic pressure, and on the cfPWV.^71^ Table 3 shows the effects of different classes of anti-hypertensive drugs on central hemodynamics.

**Table 3:** Comparative effect of different classes of anti-hypertensive drugs on central hemodynamics

Classes of anti-hypertensive drugs	CSaP	CDaP	Amplification	Reflection	cfPWV	PAP
Beta-blockers	**↑↑**	**←→**	**↓**	**↑**	**←→**	**↓**
Calcium channel blockers	**↓**	**↓/←→**	**↑**	**↓**	**↓**	**↓**
Angiotensin-converting enzyme inhibitors	**↓↓**	**↓**	**↑**	**↓**	**↓**	**↓**
Angiotensin II AT1 receptor blockers	**↓**	**↓/←→**	**↑/←→**	**↓**	**↓**	**↓**
Diuretics	**←→**	**←→**	**←→/↓**	**←→**	**←→**	**↓**
Nitrates	**↓**	**↓**	**↓**	**↓**	**←→**	**←→/↓**

CSaP: central systolic aortic pressure; CDaP: central diastolic aortic pressure; cfPWV: carotid femoral pulse wave velocity; PAP: peripheral arterial pressure.

### Beta-blockers

The CAFE study compared the effect of beta-blockers on the central pressure for a similar peripheral BP, and the atenolol/thiazide group showed higher aortic central pressure values ​​when compared with the amlodipine/perindopril group.^66^

Nebivolol (a beta-blocker with a vasodilatory effect) and carvedilol (an anti-hypertensive with alpha- and beta-blocking effects) compared with atenolol promoted a greater reduction in central aortic pressure and pulse amplification.^72,73^ Nebivolol reduces central aortic pressure and the augmentation index in mildly hypertensive patients after 3 months of treatment.^74^

### Calcium channel blockers

Calcium channel blockers reduce oxidative stress in experimental models and decrease central aortic pressure.^66^ The AORTA study compared the addition of azelnidipine or amlodipine to hypertensive patients using olmesartan and demonstrated that the azelnidipine group achieved a greater reduction in central aortic pressure and in the augmentation index, and a greater regression in left ventricular hypertrophy and left ventricular diastolic dysfunction.^75,76^

### Angiotensin converting enzyme inhibitors

The reduction in central aortic pressure demonstrated in comparative studies with angiotensin converting enzyme inhibitors (ACEi) can be attributed to possible mechanisms involving reduction in compliance and oxidative stress, structural remodeling of the vascular wall, collagen/elastin relationship, anti-inflammatory effect and consequent relaxation of the vascular smooth muscle.^77,78^

### Angiotensin II AT1 receptor blockers

Valsartan and captopril reduce to a similar extent the central aortic pressure and the cfPWV.^79^ The EXPLOR study compared valsartan/amlodipine *versus* amlodipine/atenolol for a similar BP reduction in the peripheral artery. Central aortic pressure and cfPWV showed a greater reduction in the valsartan/amlodipine group.^80^ Studies with other AT1 receptor blockers have shown similar results.^81,82^

### Diuretics

Diuretics appear to have no beneficial effect on central hemodynamics.^83,84^

### Nitrates

The effects of nitrates on central aortic pressure are attributed to the relaxation of the vascular smooth muscle of medium-caliber arteries that result in a reduction in the reflection wave amplitude, a reduction in the pulse wave velocity, and an increase in the effective reflection distance. Isosorbide mononitrate has also been evaluated in hypertensive patients and demonstrated a greater reduction in central aortic pressure than in peripheral BP and a greater reduction in the augmentation index without a significant change in the heart rate. On the other hand, nitrates do not influence cfPWV.
